# Development and Evaluation of the Magnetic Properties of a New Manganese (II) Complex: A Potential MRI Contrast Agent

**DOI:** 10.3390/ijms24043461

**Published:** 2023-02-09

**Authors:** Giovanni Reale, Francesca Calderoni, Teresa Ghirardi, Francesca Porto, Federica Illuminati, Lorenza Marvelli, Petra Martini, Licia Uccelli, Eugenia Tonini, Lucia Del Bianco, Federico Spizzo, Martina Capozza, Emiliano Cazzola, Aldo Carnevale, Melchiore Giganti, Alessandro Turra, Juan Esposito, Alessandra Boschi

**Affiliations:** 1 Department of Translational Medicine, University of Ferrara, 44121 Ferrara, Italy; 2 Medical Physics Unit, University Hospital of Ferrara, 44124 Cona, Italy; 3 Legnaro National Laboratories (LNL-INFN), National Institute of Nuclear Physics, 35020 Padua, Italy; 4 Department of Chemical, Pharmaceutical and Agricultural Sciences , University of Ferrara, 44121 Ferrara, Italy; 5 Radiology Unit, University Hospital of Ferrara, 44124 Cona, Italy; 6 Department of Environmental and Prevention Sciences, University of Ferrara, 44121 Ferrara, Italy; 7 Department of Physics and Earth Science, University of Ferrara, 44122 Ferrara, Italy; 8 Department of Molecular Biotechnologies and Health Sciences, University of Torino, 10126 Torino, Italy; 9 IRCCS Sacro Cuore Don Calabria Hospital, Negrar di Valpolicella (VR), 37024 Negrar, Italy

**Keywords:** magnetic resonance imaging, manganese, dithiocarbamates

## Abstract

Magnetic resonance imaging (MRI) is a non-invasive powerful modern clinical technique that is extensively used for the high-resolution imaging of soft tissues. To obtain high-definition pictures of tissues or of the whole organism this technique is enhanced by the use of contrast agents. Gadolinium-based contrast agents have an excellent safety profile. However, over the last two decades, some specific concerns have surfaced. Mn(II) has different favorable physicochemical characteristics and a good toxicity profile, which makes it a good alternative to the Gd(III)-based MRI contrast agents currently used in clinics. Mn(II)-disubstituted symmetrical complexes containing dithiocarbamates ligands were prepared under a nitrogen atmosphere. The magnetic measurements on Mn complexes were carried out with MRI phantom measurements at 1.5 T with a clinical magnetic resonance. Relaxivity values, contrast, and stability were evaluated by appropriate sequences. Studies conducted to evaluate the properties of paramagnetic imaging in water using a clinical magnetic resonance showed that the contrast, produced by the complex [Mn(II)(L’)_2_] × 2H_2_O (L’ = 1.4-dioxa-8-azaspiro[4.5]decane-8-carbodithioate), is comparable to that produced by gadolinium complexes currently used in medicine as a paramagnetic contrast agent.

## 1. Introduction

Magnetic resonance imaging (MRI) is a powerful non-invasive and efficient imaging technique widely used in diagnostic clinical medicine and biomedical research. In MRI, the nuclear magnetic resonance signal of protons, mainly from water, is converted into an image. To obtain high-definition pictures of tissues or of the whole organism this technique is enhanced by the use of contrast agents (CAs).

The efficiency of the metallic CAs depends on their longitudinal (r1) and transverse (r2) relaxivity, which is defined as the increase in the nuclear relaxation rate (the reciprocal of the relaxation time T1 and T2, respectively), of water protons produced by one mmol/L of CA. Therefore, the characteristic quality of an MRI CA is typically measured by the parameters of relaxivity, r1 or r2 describing the CAs ability to shorten the T1 or T2 relaxation time of water. Paramagnetic metals shorten the longitudinal relaxation time (T1) and hence increase the relaxation rate (1/T1) of solvent water protons. Accumulating organs become bright in a weighted T1 (T1*w*) MRI sequence. Therefore, these CAs are typically referred to as positive contrast media. Superparamagnetic iron oxides are regarded as negative contrast agents that influence the signal intensity mainly by shortening transverse relaxation time (T2) and producing the darkening of the weighted T2 (T2*w*) contrast-enhanced tissue [1].

Gadolinium-based contrast agents (GBCAs) have an excellent safety profile. However, over the last two decades, some specific concerns have surfaced [2]. Emerging evidence has linked MRI signal changes in deep nuclei of the brain with repeated administration of GBCAs. Accumulation of gadolinium was observed in brain tissue, especially in the dentate nuclei and globus pallidus [3]. GBCAs are connected to nephrogenic systemic fibrosis (NSF) and tissue retention of gadolinium. The GBCA safety profile debate has recently been reopened due to recent observations that have demonstrated deposition and retention in the brain; while it is known that gadolinium retention occurs in the liver and bones [2]. Many studies have highlighted the accumulation of gadolinium in the brain of adults and children, depending on the concentration administered and observed thanks to the high signal intensity in Globus pallidus and in the dentate nucleus on T1 (T1*w*) unweighted images. Post-mortem human or animal studies have validated gadolinium deposition in these T1-hyperintensity areas, raising new worries about the safety of GBCAs [4]. In vitro preclinical studies have shown that gadolinium is neurotoxic through multiple mechanisms, mainly involving Ca^2+^ homeostasis and mitochondrial functions, however, no cause-related effect has been demonstrated in patients between exposure to cerebral gadolinium and clinical consequences specific to neurological toxicity [5]. NSF is a rare fibrosing disorder with a poor prognosis, which is characterized by skin and subcutaneous thickening as well as systemic manifestations. The disease has been reported exclusively in patients with advanced renal disease, and it is associated with higher doses and specific types of GBCAs [2]. The development of NSF is strongly connected to gadodiamide administration in the setting of either acute hepatorenal syndrome or dialysis-dependent chronic renal insufficiency [6].

Manganese (Mn) was one of the earliest reported examples of paramagnetic contrast material for MRI because of its efficient positive contrast enhancement. When used as a CA, manganese ion (Mn^2+^) works similarly to other paramagnetic ions, such as gadolinium (Gd^3+^) and copper (Cu^2+^), which are capable of shortening the T1 of water protons, thus increasing the signal intensity of T1*w* MR images. Generally, Mn also has a minor T2 effect, which reduces the signal intensity to produce dark signals. At a biochemical level, manganese is involved in mitochondrial function, and the greater the mitochondria density in the cell, the higher the level of Mn uptake [1]. As opposed to gadolinium, which is a toxic element, Mn in small amounts is an essential element already present in the human body [7]. Although small amounts are essential to human health, overexposure to manganese in its free form is toxic and may result in a neurodegenerative disorder known as *manganism* with symptoms resembling Parkinson’s disease, and it needs to be “masked” before administration. Mn chelates prevent the premature release of the metal and enhance the T1 signal [2]. One of the main challenges with manganese is that it does not always stay in the chelate form to create a signal from the MRI scan [7]. Until today only one Mn(II) chelate [Mn(II)(DPDP)]^4−^ (Teslascan) was introduced for clinical use as a hepatobiliary CA for MRI. In this complex, the Mn^2+^ is chelated by a dipyridoxyldiphosphate ligand (DPDP) [8]. Sadly, after intravenous administration, [Mn(II)(DPDP)]^4−^ releases Mn^2+^ ions into the blood which are subsequently taken up by hepatocytes. Therefore, due to poor clinical performance, and concerns over toxicity, Teslascan was withdrawn from the market [9].

Several Mn-chelates have been investigated [10,11]. Among the most studied ligands is the acyclic ligand EDTA (2,2′,2′′,2′′′-(Ethane-1,2-diyldinitrilo)tetraacetic acid), which coordinates manganese to give the seven-coordinated [Mn(EDTA)(H_2_O)]^2−^ complex with relaxivity (r1) of 3.3 mM^−1^ s^−1^ at 20 MHz and 25 °C. EDTA derivates have been also developed introducing a small functional group able to reversibly interact with macromolecules. This, reducing the tumbling rate of the paramagnetic compound, seems to increase the relaxivity (r1). Many [Mn(EDTA)]^2−^ derivatives with a high relaxivity value have given promising results for MRI imaging purposes, however, they show very poor kinetic inertness due to the flexible structure of the ligand [10]. The use of macrocylic ligands such as DOTA (2,2′,2′′,2′′′-(1,4,7,10-tetraazacyclododecane-1,4,7,10-tetrayl)tetraacetic acid), NOTA (2,2′,2′′-(1,4,7-triazacyclononane-1,4,7-triyl)triacetic acid) and cyclen (1,4,7,10-tetraazacyclododecane) derivates increase the kinetic inertness of the resulting Mn coordinated complex; unfortunately, they show low relaxivity values due to the absence of inner-sphere water molecules.

We recently investigated a new class of Mn(II)-dithiocarbamates complexes to be potentially used as MRI contrast media. Dithiocarbamates (−S_2_CNR_2_, R = organic functional group) are bidentate chelating ligands, forming stable complexes with transition elements with excellent properties and potential applications [12]. It is worth noting that, while technetium-99m and rhenium-188 dithiocarbamate mononuclear compounds have found application in nuclear medicine as imaging and therapeutic agents, respectively [13,14], manganese dithiocarbamates complexes, to our knowledge, have never been studied for medical applications.

These investigations were borne within the METRICS project (Multimodal pET/mRi Imaging with Cyclotron-produced ^51/52^Mn iSotopes), which aim to produce ^52/51^Mn-radiopharmaceuticals and analogous paramagnetic complexes of manganese, for PET/MRI multimodal diagnostics applications, (PET = positron emission tomography, MRI = magnetic resonance imaging). In particular, we found that the mononuclear complex [Mn(II)L_2_] (abbreviated MnL_2_, where L = diethyldithiocarbamate anion, [S_2_CNEt_2_]− (IUPAC name: diethylcarbamodithioate) or bis(N-ethoxyethyl)dithiocarbamate (IUPAC name: bis(2-ethoxyethyl)carbamodithioate)) could only be prepared under strictly nitrogen-controlled conditions, and when in contact with air, they oxidize to Mn(III) compounds [15]. On the contrary, using the anion L’ = 1.4-dioxa-8-azaspiro[4.5]decane-8-carbodithioate, we isolated the mononuclear compound [Mn(II)(L’)_2_] × 2H_2_O, which is stable in air.

In this paper, we reported the synthesis, characterization, and evaluation of magnetic properties by phantom image studies of the new [Mn(II)(L’)_2_] × 2H_2_O compound potentially useful as MRI CA.

## 2. Results and Discussion

### 2.1. Synthesis and Characterization of Mn Complexes

The manganese complexes [Mn(II)(PTA)(Cl)_2_(H_2_O)_2_] (Mn2) and [Mn(II)(C_7_H_12_O_2_-N-C(=S)S)_2_] (Mn3) were prepared and characterized as described in the literature [15,16,17]. Briefly, all compounds were prepared by reacting the Mn(II)Cl_2_·4H_2_O (Mn1) with the PTA or dithiocarbamate ligands, respectively. The synthesis of Mn3 has been carried out under inert conditions by reacting the Mn(II) salt with two equivalents of the dithiocarbamate ligand Na[(CH_3_CH_2_OCH_2_CH_2_)_2_-N-C(=S)S]·3H_2_O) by Schlenk technique.

The synthesis of the mononuclear complex [Mn(II)(L’)_2_]·2H_2_O (Mn4), where L’ = 1.4-dioxa-8-azaspiro[4.5]decane-8-carbodithioate, was also conducted under inert conditions by reacting the Mn(II)Cl_2_·4H_2_O complex with two equivalents of the sodium salts of the ligand L’ after careful nitrogen purging of all the reaction flasks and water solvent and using the Schlenk technique. The complex was characterized by elemental, infrared (IR), and magnetometric analyses. In the IR spectra (Figure S1) the single sharp band of high intensity observed at 1004.6 cm^−1^ suggests a symmetrical bidentate binding of the dithiocarbamate moiety [18].

**Figure 1:**
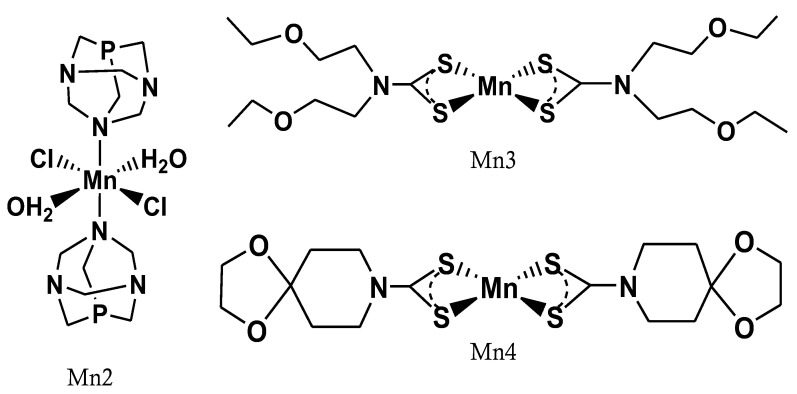
Chemical structure of the manganese compounds.

In Figure 1, the chemical structures of Mn compounds are reported.

**Figure 2:**
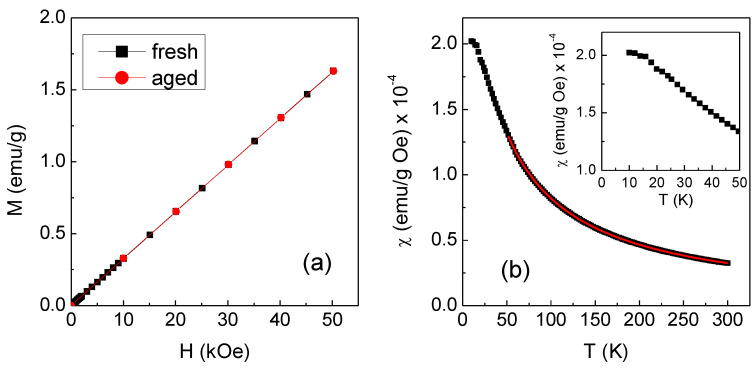
(**a**) Magnetization (M) vs. magnetic field (H) measured at T = 300 K on the freshly prepared Mn4 complex (black line) and after 7 days of aging (red line). (**b**) Mass susceptibility χ as a function of temperature T measured on the fresh Mn4 sample in H_appl_ = 10 kOe (solid symbols); the red line is the fitting curve to the Curie Weiss law. The inset is a close-up of the low-temperature region.

The magnetic properties of the Mn4 complex in powder form were analyzed by SQUID magnetometer. The measurement of the magnetization M as a function of the magnetic field H at T = 300 K was carried out on the freshly prepared sample and then repeated over time on the same sample kept in the open air. In Figure 2a, the black line is the result for the fresh sample, clearly consistent with a paramagnetic behavior; the red line, perfectly superposed on the black one, was measured after 7 days and demonstrates the high stability of the complex. The slope of the curves gives the mass susceptibility, which is defined as χ = M/H and is equal to (3.2 ± 0.1) × 10^−5^ emu/g Oe.

The magnetization M as a function of temperature T was measured in the range 10–300 K, under an applied magnetic field H_appl_ = 10 kOe. The result is shown in Figure 2b in terms of χ vs. T (black symbols). The value of χ at T = 300 K corresponds to that estimated from the M vs. H measurement. With decreasing temperature, χ increases and its trend is expected to follow the Curie Weiss law for paramagnetic materials, which is written as [19]:

(1) $$\chi =\frac{0.125}{\mathrm{MW}}\frac{\mu_{\mathrm{eff}}^{2}}{(T-\theta )}$$

where MW is the molecular weight, μ_eff_ is the effective magnetic moment per paramagnetic molecule (it is expressed in Bohr magneton unit, BM), and θ is a parameter with the dimension of a temperature.

Since a molecule of the investigated complex contains one paramagnetic Mn ion, μ_eff_ coincides with the magnetic moment per Mn ion and, based on Equation (1), is given by:

(2) $$\mu_{\mathrm{eff}}=\;2.828{\left[\chi \;\mathrm{MW}\;(T-\theta )\right]}^{1/2}$$

To estimate μ_eff_ using the above equation, the value of θ was obtained by fitting the χ vs. T curve in Figure 2b to the Curie Weiss law in the 50–300 K interval (red line in Figure 2b). The obtained value of θ was (−32 ± 1) K, the negative sign indicating the existence of magnetic interactions of antiferromagnetic type between the molecular moments. It is to be noted that for T tending to zero, the susceptibility approaches a plateau value (inset of Figure 2b), which suggests that the magnetic interactions became so strong at very low temperatures that the sample no longer behaved as a bonafide paramagnetic material.

The μ_eff_ value calculated from Equation (2) was (6.5 ± 0.4) BM (the used MW was 491.6 g/mol). The diamagnetic contribution to χ given by the elements in the complex other than Mn is lower than the estimated experimental error. It is to be noted that the same type of magnetic analysis was also conducted on the Mn1 compound, used as reference material (Figure S2). In that case, μ_eff_ = (5.9 ± 0.2) BM, in excellent agreement with that predicted for the Mn^2+^ ion in the high-spin state (i.e., 5.92 BM). Hence, even considering the experimental error, the value of μ_eff_ found for the Mn4 complex slightly exceeds that expected for the Mn^2+^ ion, probably due to residual traces of the initial reagents in the sample. We obtained values of μ_eff_ consistent with the high-spin state of Mn^2+^ also for Mn2 and Mn3 [17].

The Mn compounds were preliminarily subjected to relaxivity measurements on a Micro MR imaging and analyzing system. The ability of proton relaxation enhancement of a paramagnetic compound is commonly expressed by the term relaxivity r1 or r2, which are defined as the slope of Equation (3) in the units of mM^−1^·s^−1^:

(3) $$\left(1|T1,2\right)obs=\left(1|T1,2\right)d+r1,2\left[M\right]$$

where (1/*T*1,2)*obs* and (1/*T*1,2)*d* are water proton relaxation rates in the presence and absence of the paramagnetic species, and [*M*] is the concentration of the paramagnetic species.

Relaxivity values measured at 25 °C in two magnetic fields of 0.47 T (20 MHz) and 1.4 T (60 MHz) are reported in Table 1. As predicted by the theory of paramagnetic relaxation, it can be observed that the increase in the magnetic field determines a decrease in r1 and an increase in r2 [20].

In particular, the r1 value obtained for the Mn4 compound is higher than most of the Mn complexes reported in the literature, which it is in most cases between 1.5–5.2 mM^−1^·s^−1^ (r1) [10].

### 2.2. Phantom Studies

Based on the Mn4 promising results obtained with the Micro MR imaging system, a more detailed study was conducted collecting magnetic resonance imaging of a phantom especially realized to compare the paramagnetic contrast properties of Gadobenato dimeglumina (3,6,9-triaza-12-oxa-3,6,9-tricarboxymethylene-10-carboxy-13-phenyltridecanoic acid, gadolinium Gd-BOPTA) and Mn1, Mn2, Mn3, Mn4 compounds.

**Figure 3:**
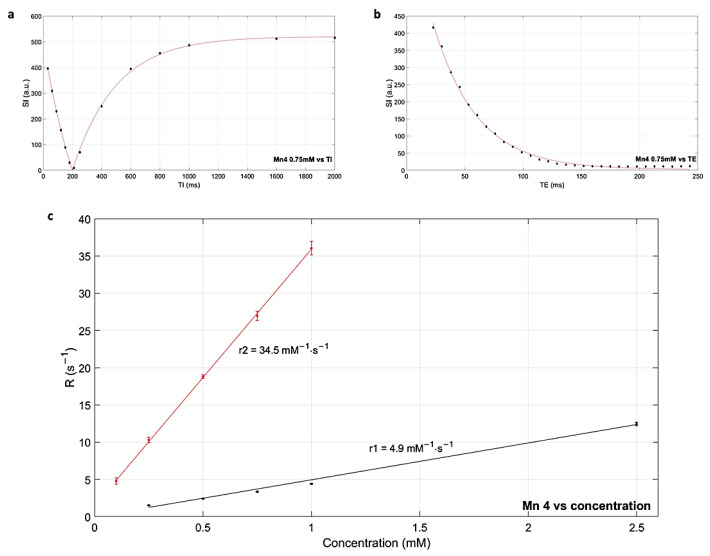
(**a**,**b**) Relaxation curves obtained for Mn4 at 0.75 mM: experimental values of mean signal intensity (SI) versus inversion time (TI) or echo time (TE) and corresponding fitting. (**c**) Determination of r1 (black) and r2 (red) for Mn4: experimental values of relaxation rates versus concentration and corresponding linear regression.

The longitudinal (T1) and transverse (T2) relaxation times of the water with Gd-BOPTA**,** Mn1, Mn2, Mn3, and Mn4 compounds were measured using inversion recovery turbo spin echo (IR TSE) and multiecho spin echo (ME-SE) sequences in 1.5 T MR clinical machine at 37 °C. Relaxation times were measured at different concentrations (Figure 3a,b) in the range allowed by sequence parameters, then the longitudinal relaxivities (r1 and r2) were obtained (Figure 3c) from the plot of relaxation rates (R1 = 1/T1 and R2 = 1/T2) versus complexes concentration. In Table 2 r1, r2, and r2/r1 values of the compounds are reported.

The r1 value of Mn4 was found to be 4.9 mM^−1^·s^−1^ at 1.5 T, which was similar to the values obtained for the Mn(II) unchelated complexes Mn1, Mn2, and higher than that of the commercially available Gadolinium MRI CA. The transverse relaxivity of Mn4 was estimated at 34.5 mM^−1^·s^−1^, almost six times greater than for Gd-BOPTA even if lower than for Mn1 and Mn2. The obtained r2/r1 is higher than the almost 1 ratio of Gd-based CAs, the consequence is the appearance of T2-related signal loss at high concentrations, narrowing the useful concentration range [21]. Anyway, in clinical practice, sequence parameters should be optimized to obtain maximum contrast [22].

Relaxivity values for Mn3 are lower than for Mn4 and in general for the Mn(II) complexes. This is in agreement with the previous observations obtained on the [Mn(II)(L)_2_] complexes that, in solution, tend to oxidize to Mn(III) complexes with a consequent loss of paramagnetic properties. On the contrary, the complex Mn4 maintains the paramagnetic properties typical of Mn(II) complexes even in solution.

Since the Mn3 and Mn4 complexes are insoluble in water, cyclodextrin has been used for their solubilization. A series of samples containing only water with TriMethyl-β-cyclodextrin at different concentrations were also inserted in the phantom and T1 e T2 of the cyclodextrin solutions were found to be in the same order of magnitude of water.

Since the presence of TriMethyl-β-cyclodextrin helps the solubilization of the complexes, it is not possible to exclude an interaction between the two species which could influence the relaxivity results. Unfortunately, the evaluation of the influence of cyclodextrin on the relaxivity values cannot be evaluated since it was not possible to carry out the relaxivity measurements in aqueous solution in the absence of the solubilizer, due to the precipitation of the Mn3 and Mn4 complexes. Although cyclodextrins are biocompounds compatible with in vivo injection and widely used as solubilizers in pharmacies, toxicity tests will have to be performed for in vivo applications.

The effectiveness of the different Mn complexes was also evaluated on sequences routinely used for abdominal contrast-enhanced MRI studies in diagnostic radiology in terms of contrast to noise ratio (CNR).

**Figure 4:**
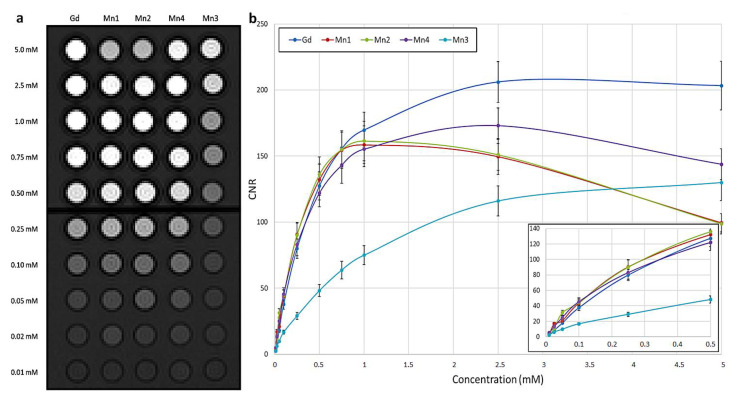
Effectiveness of Mn complexes on a T1 weighted sequence routinely used for abdominal contrast-enhanced MRI studies. (**a**) VIBE image of the phantom with glass tubes containing different complexes (Gd-BOPTA, Mn1, Mn2, Mn4, Mn3) in water at concentrations 0.01–5.0 mM. (**b**) For each complex, the contrast to noise ratio (CNR) is plotted as a function of concentrations. In the inset, a detail of low concentrations is reported.

For T1*w* imaging a VIBE (volumetric interpolated breath-hold examination) sequence was used. In addition to qualitative evaluation, the CNR was plotted as a function of concentration for each compound (Figure 4). Excluding Mn3 which presents a worse performance, Mn complexes show comparable or even higher CNR values than Gd as concentrations decrease while the latter predominates for values greater than 0.5 mM. As reported in the literature, the maximum contrast is limited due to T2-related signal decay, depending also on sequence parameters, this effect is particularly evident for Mn1 and Mn2 which present a signal reduction at the highest concentration, related to greater r2 values [20,22]. The variation of CNR relative to Gd-BOPTA in the same concentration was also computed for each Mn complex (Figure S3). This analysis further highlights the better contrast of Mn1, Mn2, and Mn4 as the concentration decreases.

**Figure 5:**
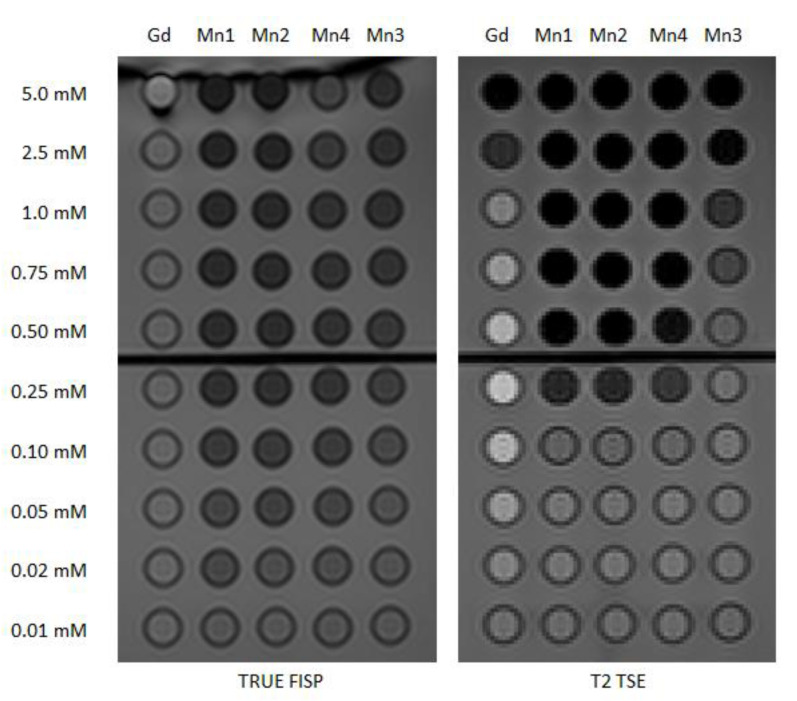
Effectiveness of Mn complexes on T2*w* sequences routinely used for abdominal contrast-enhanced MRI studies. Left: TRUE FISP image of the phantom with glass tubes containing different complexes (Gd-BOPTA, Mn1, Mn2, Mn4, Mn3) in water at concentrations 0.01–5.0 mM. Right: T2 TSE image of the same phantom.

For T2 weighted (T2*w*) imaging, TRUE FISP (true fast imaging with steady-state precession) and TSE (turbo spin echo) sequences were tested both visually (Figure 5) and considering signal and CNR values. The signal produced by the former is related to T2/T1 ratio and produces for Gd-BOPTA an intensity that is not statistically different from water, for the TSE sequence the behavior reflects the standard trend of spin echo signal for the chosen values of TR and TE, with positive or negative contrast relative to water depending on the concentration. Analyzing Mn compounds, while low concentrations have no or just a slight effect, values higher than 0.10 mM are able to strongly reduce the signal, up to one-half in the case of TRUE FISP and also more for T2 TSE.

The MRI signal stability of Gd-BOPTA, Mn1, Mn2, and Mn4 compounds for these three sequences was also evaluated. A preliminary stability study, conducted by capturing the MR images of the preparations in solution (in the concentration range of 0.50 mM–5.0 mM) for a few days, showed that in most cases there are no significant changes in the signal intensities up to 72 h (Table 3). The most sensitive sequence was the T2 TSE, with variations higher than 20% for all the Mn compounds. For in vivo studies, further insights into the stability of the complexes will need to be investigated.

## 3. Materials and Methods

Na[S_2_CN(CH_2_CH_2_OEt)_2_] and the sodium salt of the ligand L’ = 1.4-dioxa-8-azaspiro[4.5]decane-8-carbodithioate (NaL’) were obtained from Alchemy Srl, Baricella, Italy. The heterocyclic phosphine 1,3,5-triaza-7-phosphaadamantane (PTA) and the complex [Mn(II)(PTA)(Cl)_2_(H_2_O)_2_] (Mn2) were prepared according to the procedure reported in the literature [16]. Mn(II)L_2_ (Mn3) complex was prepared by the addition of MnCl_2_·4H_2_O in 5 mL Milli-Q water to two equivalents of the sodium salt of the ligand L = [C_7_H_12_O_2_-N-C(=S)S^−^], as recently reported. Gadobenato dimeglumina MultiHance 0.5 M was obtained from Bracco, Milano, Italy.

The elemental analysis (EA) was performed by means of the Thermo Scientific™ FLASH 2000 Analyzer, Thermo Fisher Scientific, Waltham, MA, USA. The infrared spectra (IR) were recorded with an FT-VERTEX 70 (Bruker, Billerica, MA, USA) in the range 4000–400 cm^−1^ using anhydrous KBr.

### 3.1. Synthesis and Characterization of [Mn(II)(L’)_2_] × 2H_2_O (Mn4)

The compound Mn4 was prepared by dropwise addition of MnCl_2_·4H_2_O (0.50 mmol, 0.100 g) in 3 mL Milli-Q water to two equivalents of the sodium salt of the ligand L’ = 1.4-dioxa-8-azaspiro[4.5]decane-8-carbodithioate (NaL’) (1.01 mmol, 0.244 g) previously dissolved in 5 mL of Milli-Q water. The preparation was conducted under oxygen-free conditions. The precipitation of a yellow product was rapidly observed which, after leaving the reaction for 30 min under magnetic stirring, was filtered and washed with water, still under nitrogen atmosphere. The isolated precipitate, if kept in a controlled atmosphere, maintains a yellow color. The compound is soluble in organic solvents such as acetonitrile, chloroform, and acetone. (Yield, 85%). Analysis calculated for C_16_H_24_O_4_N_2_S_4_Mn·2H_2_O: C, 36.4%; H, 5.3%; S, 24.3%; N, 5.3%. Found: C, 35.8%; H, 4.9%; S, 23.9%; N, 5.2%.ESI-MS: [Mn(L’)]^+^, expected m/z for C_8_H_12_O_2_N_1_S_2_Mn = 144.88; found *m*/*z* = 144.34 [M]+. FT IR: ν(O-H): 3526 cm^−1^; ν(C-N): 1475 cm^−1^; ν(C-S): 1004 cm^−1^.

### 3.2. Magnetic Measurement

The magnetic measurements were conducted using a superconducting quantum interference device (SQUID) magnetometer (Quantum Design, San Diego, CA, USA) operating in the 5–300 K temperature range in a He atmosphere of ~100 Pa (maximum applied field H = 50 kOe, sensitivity 10^−7^ emu). To calculate the specific magnetization (M = magnetic moment/sample mass, expressed in emu/g) the mass of the sample was measured with a precision of 10^−5^ g.

### 3.3. Relaxometric Investigations

A Stelar Spinmaster spectrometer (Stelar, Mede, Italy) working at the adjustable field has been used to measure the longitudinal (r1 = 1/T1) and transverse (r2 = 1/T2) relaxation rates at 25 °C at two magnetic field strengths, 0.47 T and 1.4 T (corresponding to 20 MHz and 60 MHz proton Larmor frequency). T1 values were measured using the standard inversion recovery sequence with a typical radio frequency 90° pulse length of 3.5 μs. T2 values were measured using a standard CPMG sequence (2048 sampled echoes, 16 scans, and 2 averages). The temperature was controlled with a Stelar VTC-91 heater (Stelar, Mede, Italy) airflow equipped with a copper–constantan thermocouple (uncertainty of ±0.1 K). The relaxation rates of the solutions containing the paramagnetic metal compounds were subtracted from the corresponding diamagnetic contributions and then divided by the concentration of Mn atoms to obtain the normalized millimolar relaxivities (r1 and r2, in mM^−1^·s^−1^).

### 3.4. Phantom Studies

**Figure 6:**
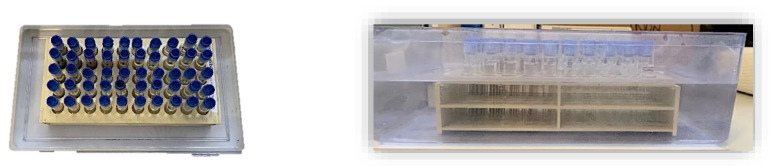
Photo of the phantom view from above on the left and transversal on the right.

The 500 mM Gadobenato dimeglumina solution was diluted with milliQ H_2_O to obtain a series of different concentrations (0.01–5.00 mM). Solutions of MnCl_2_ × 4H_2_O (Mn1) and [Mn(PTA)(Cl)_2_(H_2_O)_2_] (Mn2) compounds were prepared from a 5 mM stock solution and diluted with milliQ H_2_O. Solution of Mn3 and Mn4 compounds were prepared from a 5 mM stock solution containing 2 g of TriMethyl-β-cyclodextrin, used as a solubilizer, and diluted with milliQ H_2_O. Each solution (5 mL) was put into a glass tube (12 × 75 mm), inserted in a samples holder, and maintained airtight without bubbles. A series of samples containing only water with TriMethyl-β-cyclodextrin at different concentrations were also inserted in the phantom. The assembled tubes were immersed in a box filled with water at 37 °C (Figure 6) [23].

All MR examinations of the phantom were performed at 1.5 T (MAGNETOM Aera, Siemens Healthcare, Erlangen, Germany) with the use of an abdomen coil. The phantom study aimed to evaluate in a pre-clinical scenario the relaxivity of the new compounds under investigation and their contrast properties compared to Gadobenato dimeglumina, both quantitatively and qualitatively. For this reason, two separate MRI protocol sections were implemented:

- The first part of the protocol was a modified version of the protocol described by Rohrer et al. [24] Coronal T1 IR TSE and T2 ME-SE were obtained. T1 IR TSE parameters were TR = 3000 ms, TE = 7.3 ms, TI = 30-60-90-120-150-180-210-250-400-600-800-1000-1600-2000 ms, ETL = 3, FA = 180, voxel = 1.2 mm × 1.2 mm × 3.0 mm, FOV = 300 mm, matrix = 256 × 256. T2 ME-SE parameters were TR = 3000 ms, echo spacing = 7.6 ms, ETL = 32, FA = 180, voxel = 0.8 mm × 0.8 mm × 3.0 mm, FOV = 300 mm, matrix = 384 × 384;
- The second part of the protocol included sequences routinely used for abdominal contrast-enhanced MRI studies in diagnostic radiology. Manganese is an excellent contrast agent for MR imaging of the liver and similar mitochondria-rich organs such as the pancreas and kidneys, as reported by Pan et al. [1]. On the other hand, Gadobenate dimeglumine (also known as MultiHanceTM) is an extracellular intravenous contrast agent used in clinical MRI, and it can be useful in a wide range of MRI applications, including hepatic imaging, pelvic imaging and it can also be used as a hepatobiliary phase agent [25]. Given these two main considerations, it was therefore decided to complete the phantom study with this clinically oriented section, inspired by a standard abdominal protocol. Coronal T1 VIBE, T2 TSE, and TRUE FISP with default vendors parameters were obtained. T1 VIBE parameters were TR = 5.06 ms, TE = 2.43 ms, ETL = 1, FA = 10, voxel = 1.2 mm × 1.2 mm × 3.0 mm, FOV = 300 mm, matrix = 256 × 256. T2 TSE parameters were TR = 3890 ms, TE = 112 ms, ETL = 29, FA = 180, voxel = 1.2 mm × 1.3 mm × 3.0 mm, FOV = 320 mm, matrix = 224 × 256. TRUE FISP parameters were TR = 1077.36 ms, TE = 2.29 ms, ETL = 1, FA = 60, voxel = 0.7 mm × 0.7 mm × 3.0 mm, FOV = 300 mm, matrix = 448 × 448.

The images obtained were quantitatively analyzed with the software ImageJ (ImageJ 1.44o, U.S. National Institutes of Health, Bethesda, MD, USA) and Matlab (MathWorks, Natick, MA, USA). *T*1 and *T*2 values were obtained fitting experimental mean signal intensities (SIs) through Equations (4) and (5), respectively, and fitting errors were used to describe relaxation times uncertainties [26].

(4) $$SI\left(TI\right)=A_{1}\left(1-B_{1}e^{-\frac{TI}{T1}}\right)$$

(5) $$SI\left(TE\right)=A_{2}e^{-\frac{TE}{T2}}+B_{2}$$

By definition, relaxivities were estimated from Equation (3) through the linear regression of experimental relaxation rates (1/*T*1 and 1/*T*2) as a function of complexes concentration (C).

To quantitatively evaluate the contrast of the different complexes and concentrations in sequences routinely used for abdominal contrast-enhanced MRI studies, the contrast-to-noise ratio (CNR) was used. This value was computed as in Equation (6) considering the mean signal intensity and the standard deviation (SD) within regions of interest placed into analyzed complex and background (BKG).

(6) $$CNR=\frac{SI_{complex}-SI_{BKG}}{SD_{BKG}}$$

## 4. Conclusions

With the aim to develop Mn-based CAs, we recently investigated a new class of Mn(II)-dithiocarbamates complexes to be potentially used as MRI contrast media.

In particular, we found that the mononuclear complexes [Mn(II)(L)_2_], where L = anion diethyldithiocarbammate or bis(N-ethoxyethyl)dithiocarbamate can be prepared under controlled conditions in the absence of oxygen. Unfortunately, when in contact with air, they oxidize to Mn(III) compounds by decreasing the paramagnetic properties typical of Mn(II) compounds. On the contrary, using the anion L’ = 1.4-dioxa-8-azaspiro[4.5]decane-8-carbodithioate, it was possible to isolate the mononuclear compound [Mn(II)(L’)2] × 2H_2_O, which is stable in air. This observation suggests that the greater rigidity and/or steric hindrance of the azaspiro moiety on the nitrogen atom of the carbamate, replacing aliphatic groups (such as Et, EtOCH_2_CH_2_-) seems to stabilize the compound of Mn(II)(L’)_2_. Studies conducted to evaluate the properties of paramagnetic imaging in water using a clinical magnetic resonance showed that the contrast produced by the complex [Mn(II)(L’)_2_] × 2H_2_O is comparable to that produced by gadolinium complexes currently used in medicine as a paramagnetic contrast agent. Stability and cytotoxicity together with labeling studies are in progress.

## Figures and Tables

**Table 1:** r1 and r2 of manganese compounds.

Compound	r1 (mM^−1^·s^−1^)		r2 (mM^−1^·s^−1^)	
	20 MHz	60 MHz	20 MHz	60 MHz
Mn2	4.86 ± 0.07	2.40 ± 0.05	-	-
Mn3	2.55 ± 0.05	2.35 ± 0.03	11.01 ± 0.78	22.55 ± 0.48
Mn4	9.63 ± 0.04	7.91 ± 0.07	31.15 ± 0.12	48.31 ± 0.05

**Table 2:** r1 and r2 values of gadolinium and manganese compounds together with R^2^ coefficients. For each compound, the corresponding r2/r1 ratio is also reported.

Compound	r1 (mM^−1^·s^−1^)	R^2^	r2 (mM^−1^·s^−1^)	R^2^	r2/r1
Gd-BOPTA	4.0 ± 0.1	0.999	5.8 ± 0.1	0.999	1.5
Mn1	4.5 ± 0.1	0.999	62.6 ± 1.0	0.994	13.9
Mn2	4.4 ± 0.1	0.999	63.3 ± 0.8	0.996	14.4
Mn4	4.9 ± 0.2	0.998	34.5 ± 0.4	0.999	7.0
Mn3	1.2 ± 0.1	0.998	5.9 ± 0.7	0.971	4.9

**Table 3:** Evaluation of the stability of signal intensities for repeated examinations 72 h apart. Results are represented through color code for each combination of sequence–compound–concentration: in green variations up to 10%, in yellow from 10% to 20%, and in red higher values.

VIBE															
Gd-BOPTA				Mn1				Mn2				Mn4			
5.0	2.5	1.0	0.5	5.0	2.5	1.0	0.5	5.0	2.5	1.0	0.5	5.0	2.5	1.0	0.5
T2 TSE															
Gd-BOPTA				Mn1				Mn2				Mn4			
5.0	2.5	1.0	0.5	5.0	2.5	1.0	0.5	5.0	2.5	1.0	0.5	5.0	2.5	1.0	0.5
TRUE FISP															
Gd-BOPTA				Mn1				Mn2				Mn4			
5.0	2.5	1.0	0.5	5.0	2.5	1.0	0.5	5.0	2.5	1.0	0.5	5.0	2.5	1.0	0.5

## Data Availability

Not applicable.

## Supplementary Materials

The supporting information can be downloaded at: https://www.mdpi.com/article/10.3390/ijms24043461/s1.
