# Supplementary material for: Development and Evaluation of the Magnetic Properties of a New Manganese (II) Complex: A Potential MRI Contrast Agent
Source: Int J Mol Sci. 2023 Feb 9;24(4):3461. doi: 10.3390/ijms24043461 (PMC9965609; doi:10.3390/ijms24043461)
Supplement: Supplementary file 1 [file ijms-24-03461-s001.zip › ijms-2148746-supplementary.pdf]

Supplementary Materials

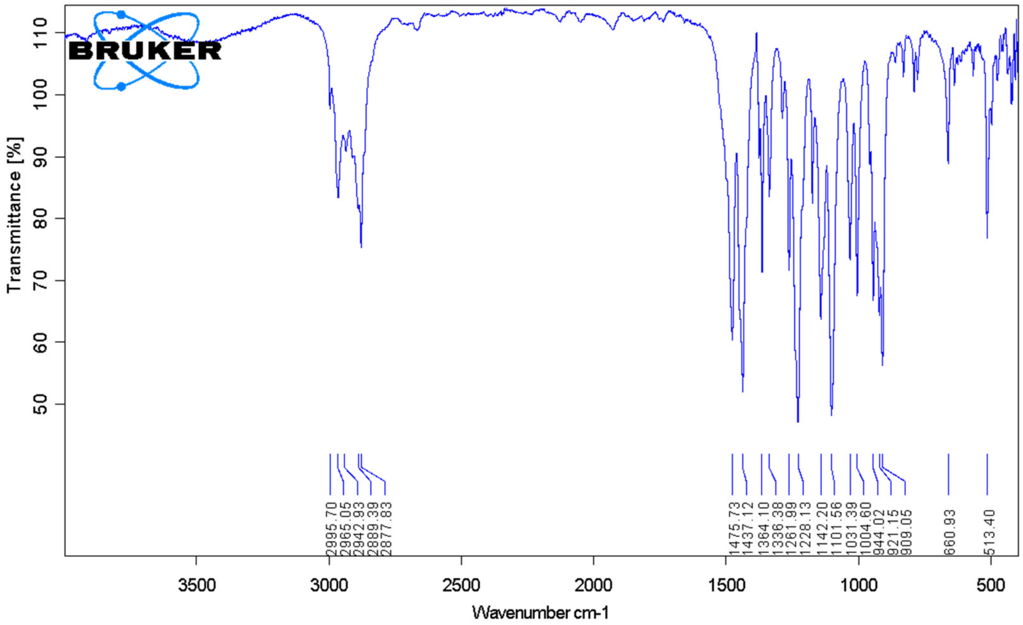

Figure S1. IR spectra of the Mn4 compound

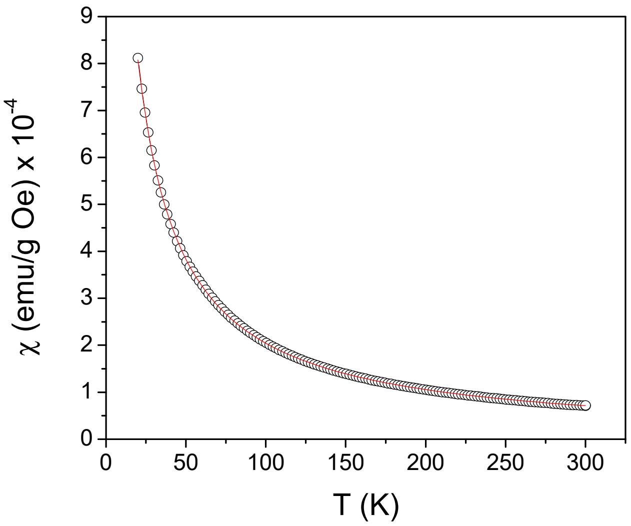

Figure S2. Mass susceptibility (χ) as a function of temperature (T) measured on the Mn1 compound, in H_{appl} = 10 kOe (open symbols). At T = 300 K, χ = (7.2 ± 0.2) $\times 10^{-5}$ emu/g Oe. The red line is the fitting curve to the Curie Weiss law with θ = (-7.0 ± 0.3) K. From relation (2) reported in the main article, μ_{eff} = (5.9 ± 0.2) BM.

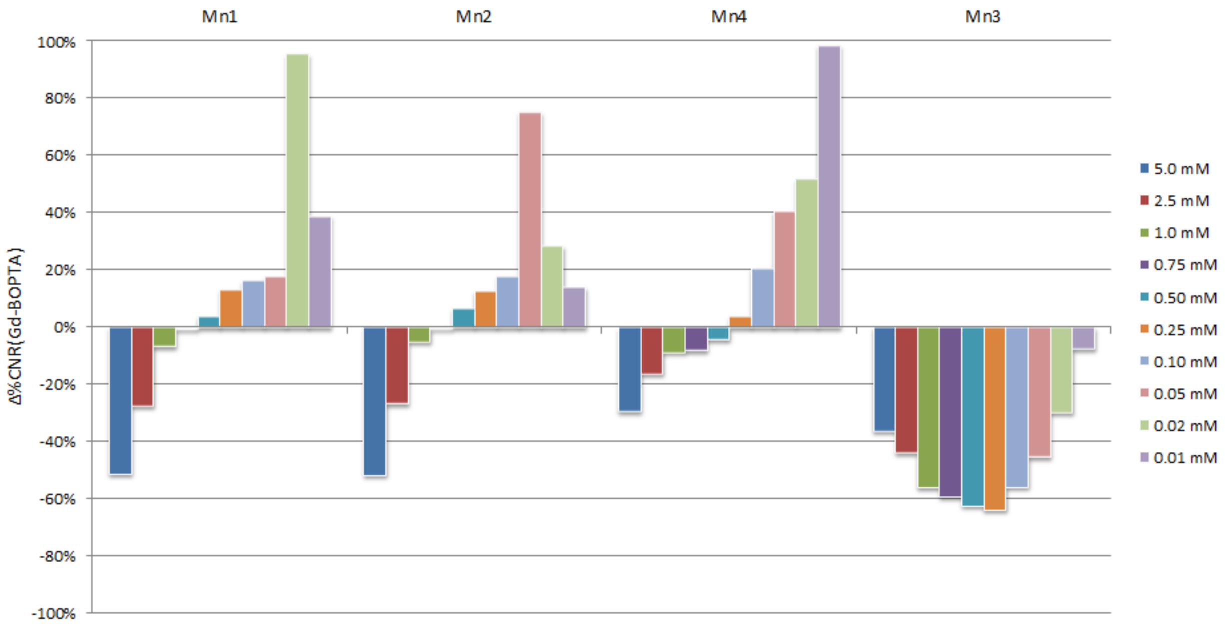

Figure S3. Variation of contrast to noise ratio (CNR) relative to Gd-BOPTA in the same concentration for Mn complexes represented in graphical format.
